# Supplementary material for: Saccharomyces boulardii (CNCM I-745) improves intestinal damage in sepsis by remodeling intestinal flora structure
Source: Front Cell Infect Microbiol. 2025 Jul 31;15:1602792. doi: 10.3389/fcimb.2025.1602792 (PMC12350329; doi:10.3389/fcimb.2025.1602792)
Supplement: Supplementary file 1 [file DataSheet1.zip › summary/summary/6_Taxonomy_community/1_Taxonomy_profiling/5_Family/Group/Family_abund_top30_Group.heatmap.pdf]

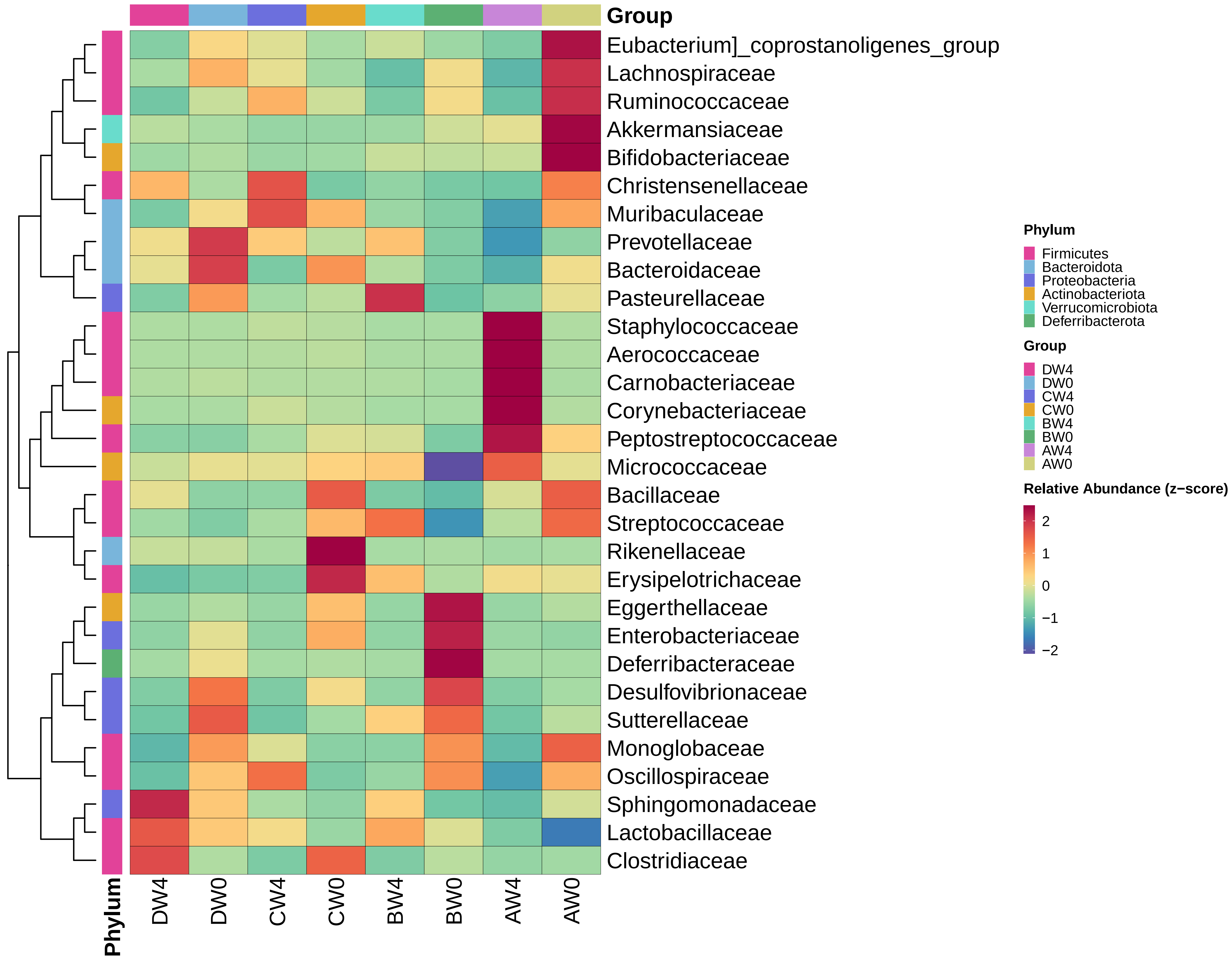
Group
Eubacterium]_coprostanoligenes_group
Lachnospiraceae
Ruminococcaceae
Akkermansiaceae
Bifidobacteriaceae
Christensenellaceae
Muribaculaceae
Prevotellaceae
Bacteroidaceae
Pasteurellaceae
Staphylococcaceae
Aerococcaceae
Carnobacteriaceae
Corynebacteriaceae
Peptostreptococcaceae
Micrococcaceae
Bacillaceae
Streptococcaceae
Rikenellaceae
Erysipelotrichaceae
Eggerthellaceae
Enterobacteriaceae
Deferribacteraceae
Desulfovibrionaceae
Sutterellaceae
Monoglobaceae
Oscillospiraceae
Sphingomonadaceae
Lactobacillaceae
Clostridiaceae
Phylum
DW4
DW0
CW4
CW0
BW4
BW0
AW4
AW0
Phylum
Firmicutes
Bacteroidota
Proteobacteria
Actinobacteriota
Verrucomicrobiota
Deferribacterota
Group
DW4
DW0
CW4
CW0
BW4
BW0
AW4
AW0
Relative Abundance (z−score)
2
1
0
−1
−2
